# *fac*-Bis(acetonitrile-κ*N*)tricarbonyl­(trifluoro­acetato-κ*O*)rhenium(I)

**DOI:** 10.1107/S1600536808029966

**Published:** 2008-09-24

**Authors:** Reza Kia, Hoong-Kun Fun

**Affiliations:** a X-ray Crystallography Unit, School of Physics, Universiti Sains Malaysia, 11800 USM, Penang, Malaysia

## Abstract

In the title compound, [Re(CF_3_COO)(CH_3_CN)_2_(CO)_3_], the Re atom has a distorted octa­hedral configuration. The two acetonitrile mol­ecules and two of the three carbonyl groups occupy the equatorial plane of the complex, with the third carbonyl ligand and the trifluoroacetato ligand in the axial positions. The three carbonyl ligands are arranged in a *fac* configuration around the Re atom. The CF_3_ segment of the trifluoroacetato ligand shows rotational disorder and the refined site-occupancy factors of the disordered parts are *ca* 0.5/0.5. The crystal structure is stabilized by C—H⋯O and C—H⋯F hydrogen bonds.

## Related literature

For values of standard bond lengths, see: Allen *et al.* (1987 ▶). For related structures, see, for example: Chan *et al.* (1977 ▶); Laza­rova *et al.* (2004 ▶). For background on the applications, see, for example: Davies & Hartely (1981 ▶); Collin & Sauvage (1989 ▶); Balzani *et al.* (1996 ▶); Meyer (1989 ▶).
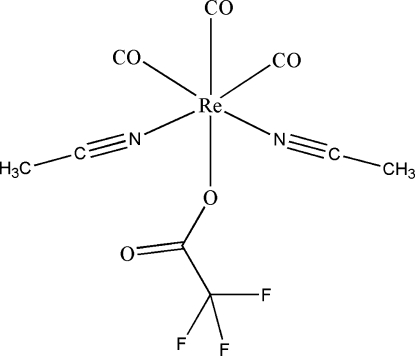


## Experimental

### 

#### Crystal data

[Re(C_2_F_3_O_2_)(C_2_H_3_N)_2_(CO)_3_]
*M* *_r_* = 465.36
Monoclinic, 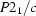

*a* = 10.8243 (2) Å
*b* = 10.4745 (2) Å
*c* = 14.4772 (3) Å
β = 125.584 (1)°
*V* = 1334.90 (4) Å^3^
*Z* = 4
Mo *K*α radiation
μ = 9.16 mm^−1^
*T* = 100.1 (1) K
0.32 × 0.23 × 0.19 mm

#### Data collection

Bruker SMART APEXII CCD area-detector diffractometer
Absorption correction: multi-scan (*SADABS*; Bruker, 2005 ▶) *T*_min_ = 0.098, *T*_max_ = 0.175
24096 measured reflections
5819 independent reflections
5087 reflections with *I* > 2σ(*I*)
*R*_int_ = 0.037

#### Refinement

*R*[*F* ^2^ > 2σ(*F* ^2^)] = 0.031
*wR*(*F* ^2^) = 0.078
*S* = 1.06
5819 reflections
211 parameters
H-atom parameters constrained
Δρ_max_ = 2.80 e Å^−3^
Δρ_min_ = −1.27 e Å^−3^

### 

Data collection: *APEX2* (Bruker, 2005 ▶); cell refinement: *APEX2*; data reduction: *SAINT* (Bruker, 2005 ▶); program(s) used to solve structure: *SHELXTL* (Sheldrick, 2008 ▶); program(s) used to refine structure: *SHELXTL*; molecular graphics: *SHELXTL*; software used to prepare material for publication: *SHELXTL* and *PLATON* (Spek, 2003 ▶).

## Supplementary Material

Crystal structure: contains datablocks global, I. DOI: 10.1107/S1600536808029966/at2632sup1.cif

Structure factors: contains datablocks I. DOI: 10.1107/S1600536808029966/at2632Isup2.hkl

Additional supplementary materials:  crystallographic information; 3D view; checkCIF report

## Figures and Tables

**Table 1:** Hydrogen-bond geometry (Å, °)

*D*—H⋯*A*	*D*—H	H⋯*A*	*D*⋯*A*	*D*—H⋯*A*
C4—H4*B*⋯O5^i^	0.98	2.42	3.113 (5)	127
C6—H6*A*⋯F1*A*^ii^	0.98	2.35	3.237 (8)	150
C6—H6*B*⋯O5^iii^	0.98	2.46	3.075 (5)	121

Symmetry codes: (i) 
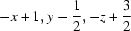
; (ii) 
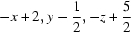
; (iii) 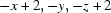.

**Fig. 1.**
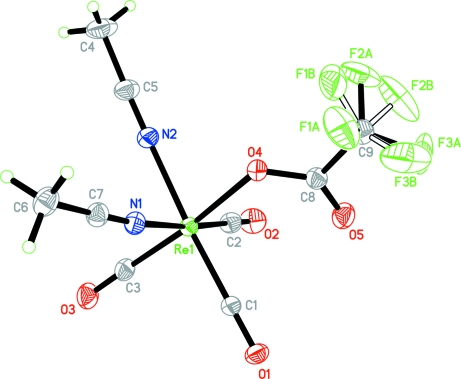
The molecular structure of the title compound, showing 40% probability displacement ellipsoids and the atomic numbering scheme. The open bonds indicate the disordered parts.

**Fig. 2.**
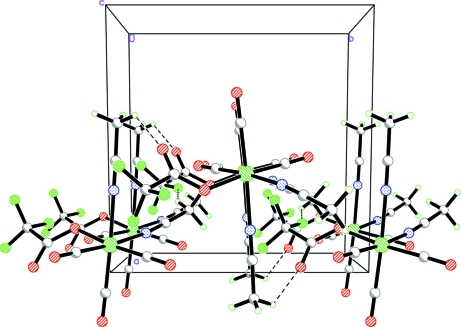
The crystal packing of the major component of (I), viewed down the c-axis. Intermolecular interactions are shown as dashed lines.

## supplementary crystallographic information

### Comment

The synthesis of solvent-coordinated complexes is a matter of considerable interest since they are useful sources for synthesis of new species resulting from the substitution of the coordinated solvent by a more basic ligand (Davies & Hartely, 1981). The lability of the solvent ligand easily gives rise to a highly reactive 16e^-^ electrophilic fragment able to activate small molecules thus providing an important step in many chemical processes. On the other hand, Rhenium tricarbonyl complexes have been the subject of much attention, mainly because of their photophysical (Meyer 1989) and photochemical properties (Collin & Sauvage 1989) and in supramolecular chemistry (Balzani *et al.*, 1996).

In the title compound (I) (Fig. 1), the Re atom adopts a distorted octahedral geometry. The bond lengths (Allen *et al.*, 1987) and angles are within the normal ranges and are comparable to the related structures (Chan *et al.*, 1977; Lazarova *et al.*, 2004). The two acetonitriles and two carbonyl groups occupy the equatorial plane of the complex, with the third carbonyl ligands and the trifluoroacetate in the axial positions. The three carbonyl ligands at Re atom are arranged in a *fac* configuration. The *cis*-equatorial angle of N1–Re1–N2 is 81.88 (13) °. The deviation of the Re atom from the C1/C2/N1/N2 plane is -0.048 (1) Å.

The crystal structure is stabilized by C—H···O and C—H···F hydrogen bonds (Table 1, Fig. 2).

### Experimental

The synthetic method has been described earlier (Chan *et al.*, 1977). Single crystals suitable for *X*-ray diffraction were obtained by evaporation of an acetonitrile solution at room temperature.

### Refinement

The hydrogen atoms of the methyl groups were first located from the difference Fourier map and then constrained to refine using a rotating-group model. The CF_3_ segment of the trifluoroacete ligand has rotational disorder and the refined site-occupancy factores of the disorder parts are 0.501 (2)/0.499 (2). The highest peak (2.80 eÅ^-3^) is located 0.61 Å from Re1 and the deepest hole (-1.27 eÅ^-3^) is located 1.01 Å from Re1.

### Crystal data

**Table:**

[Re(C_2_F_3_O_2_)(C_2_H_3_N)_2_(CO)_3_]	*F*(000) = 864
*M**_r_* = 465.36	*D*_x_ = 2.316 Mg m^−^^3^
Monoclinic, *P*2_1_/*c*	Mo *K*α radiation, λ = 0.71073 Å
Hall symbol: -P 2ybc	Cell parameters from 9896 reflections
*a* = 10.8243 (2) Å	θ = 2.6–40.1°
*b* = 10.4745 (2) Å	µ = 9.16 mm^−^^1^
*c* = 14.4772 (3) Å	*T* = 100 K
β = 125.584 (1)°	Block, colourless
*V* = 1334.90 (4) Å^3^	0.32 × 0.23 × 0.19 mm
*Z* = 4	

### Data collection

**Table:**

Bruker SMART APEXII CCD area-detector diffractometer	5819 independent reflections
Radiation source: fine-focus sealed tube	5087 reflections with *I* > 2σ(*I*)
graphite	*R*_int_ = 0.037
φ and ω scans	θ_max_ = 35.0°, θ_min_ = 2.6°
Absorption correction: multi-scan (SADABS; Bruker, 2005)	*h* = −17→14
*T*_min_ = 0.098, *T*_max_ = 0.175	*k* = −10→16
24096 measured reflections	*l* = −23→23

### Refinement

**Table:**

Refinement on *F*^2^	Primary atom site location: structure-invariant direct methods
Least-squares matrix: full	Secondary atom site location: difference Fourier map
*R*[*F*^2^ > 2σ(*F*^2^)] = 0.031	Hydrogen site location: inferred from neighbouring sites
*wR*(*F*^2^) = 0.078	H-atom parameters constrained
*S* = 1.06	*w* = 1/[σ^2^(*F*_o_^2^) + (0.0375*P*)^2^ + 1.8212*P*] where *P* = (*F*_o_^2^ + 2*F*_c_^2^)/3
5819 reflections	(Δ/σ)_max_ = 0.002
211 parameters	Δρ_max_ = 2.80 e Å^−^^3^
0 restraints	Δρ_min_ = −1.27 e Å^−^^3^

### Special details

**Table:**

Experimental. The low-temperature data was collected with the Oxford Cyrosystem Cobra low-temperature attachment.
Geometry. All esds (except the esd in the dihedral angle between two l.s. planes) are estimated using the full covariance matrix. The cell esds are taken into account individually in the estimation of esds in distances, angles and torsion angles; correlations between esds in cell parameters are only used when they are defined by crystal symmetry. An approximate (isotropic) treatment of cell esds is used for estimating esds involving l.s. planes.
Refinement. Refinement of F^2^ against ALL reflections. The weighted R-factor wR and goodness of fit S are based on F^2^, conventional R-factors R are based on F, with F set to zero for negative F^2^. The threshold expression of F^2^ > 2sigma(F^2^) is used only for calculating R-factors(gt) etc. and is not relevant to the choice of reflections for refinement. R-factors based on F^2^ are statistically about twice as large as those based on F, and R- factors based on ALL data will be even larger.

### Fractional atomic coordinates and isotropic or equivalent isotropic displacement parameters (Å^2^)

**Table:**

	*x*	*y*	*z*	*U*_iso_*/*U*_eq_	Occ. (<1)
Re1	0.681282 (13)	−0.021815 (12)	0.800806 (9)	0.02552 (4)	
F1A	0.9350 (14)	0.2534 (7)	1.1194 (5)	0.073 (4)	0.501 (19)
F2A	0.7397 (8)	0.3650 (13)	1.0179 (9)	0.075 (4)	0.501 (19)
F3A	0.938 (2)	0.4097 (12)	1.0310 (12)	0.101 (6)	0.501 (19)
F1B	0.818 (2)	0.2830 (10)	1.0906 (11)	0.096 (7)	0.499 (19)
F2B	0.7687 (18)	0.4304 (11)	0.9808 (9)	0.104 (6)	0.499 (19)
F3B	0.9837 (11)	0.358 (2)	1.0782 (13)	0.121 (8)	0.499 (19)
O1	0.9541 (3)	0.0126 (3)	0.7902 (3)	0.0368 (5)	
O2	0.5020 (4)	0.1538 (3)	0.5934 (2)	0.0477 (7)	
O3	0.5809 (4)	−0.2505 (3)	0.6407 (3)	0.0461 (6)	
O4	0.7533 (3)	0.1237 (2)	0.9276 (2)	0.0308 (4)	
O5	0.8149 (4)	0.2725 (3)	0.8489 (2)	0.0425 (6)	
N1	0.7910 (3)	−0.1433 (3)	0.9467 (2)	0.0307 (5)	
N2	0.4997 (4)	−0.0360 (3)	0.8190 (3)	0.0338 (6)	
C1	0.8537 (4)	−0.0010 (3)	0.7956 (3)	0.0284 (5)	
C2	0.5700 (4)	0.0891 (4)	0.6720 (3)	0.0353 (7)	
C3	0.6165 (4)	−0.1638 (4)	0.7004 (3)	0.0339 (6)	
C4	0.2941 (6)	−0.0491 (6)	0.8572 (5)	0.0620 (15)	
H4A	0.3436	−0.0624	0.9386	0.093*	
H4B	0.2247	−0.1201	0.8144	0.093*	
H4C	0.2368	0.0311	0.8336	0.093*	
C5	0.4088 (5)	−0.0429 (4)	0.8350 (3)	0.0401 (8)	
C6	0.8744 (5)	−0.3017 (4)	1.1107 (3)	0.0398 (7)	
H6A	0.9143	−0.2542	1.1811	0.060*	
H6B	0.9531	−0.3591	1.1211	0.060*	
H6C	0.7859	−0.3517	1.0915	0.060*	
C7	0.8301 (4)	−0.2132 (3)	1.0199 (3)	0.0327 (6)	
C8	0.8016 (4)	0.2303 (3)	0.9217 (3)	0.0322 (6)	
C9	0.8499 (5)	0.3206 (4)	1.0215 (3)	0.0405 (7)	

### Atomic displacement parameters (Å^2^)

**Table:**

	*U*^11^	*U*^22^	*U*^33^	*U*^12^	*U*^13^	*U*^23^
Re1	0.02296 (6)	0.02704 (7)	0.02680 (6)	−0.00061 (4)	0.01461 (5)	0.00002 (4)
F1A	0.093 (8)	0.062 (4)	0.035 (3)	0.002 (4)	0.020 (3)	−0.009 (2)
F2A	0.045 (3)	0.095 (8)	0.076 (6)	0.009 (4)	0.030 (4)	−0.042 (6)
F3A	0.165 (15)	0.083 (7)	0.109 (9)	−0.093 (9)	0.110 (11)	−0.062 (6)
F1B	0.173 (17)	0.072 (6)	0.102 (8)	−0.060 (9)	0.113 (11)	−0.049 (6)
F2B	0.127 (11)	0.060 (6)	0.068 (5)	0.036 (6)	0.025 (5)	−0.015 (4)
F3B	0.037 (4)	0.202 (19)	0.112 (10)	−0.039 (7)	0.037 (5)	−0.108 (12)
O1	0.0305 (13)	0.0433 (14)	0.0409 (13)	−0.0065 (10)	0.0232 (11)	−0.0090 (10)
O2	0.0454 (16)	0.0530 (17)	0.0389 (13)	0.0121 (14)	0.0212 (12)	0.0132 (12)
O3	0.0558 (18)	0.0402 (14)	0.0468 (14)	−0.0161 (13)	0.0324 (14)	−0.0126 (12)
O4	0.0333 (12)	0.0281 (10)	0.0346 (10)	−0.0033 (9)	0.0218 (10)	−0.0029 (8)
O5	0.0503 (16)	0.0378 (13)	0.0446 (13)	−0.0049 (12)	0.0306 (13)	0.0024 (11)
N1	0.0304 (13)	0.0288 (12)	0.0331 (12)	−0.0023 (10)	0.0187 (11)	−0.0003 (10)
N2	0.0314 (14)	0.0352 (14)	0.0377 (13)	−0.0035 (11)	0.0218 (12)	−0.0036 (11)
C1	0.0280 (14)	0.0268 (12)	0.0309 (13)	−0.0012 (11)	0.0175 (12)	−0.0028 (10)
C2	0.0330 (16)	0.0398 (18)	0.0342 (14)	0.0019 (13)	0.0202 (13)	0.0022 (13)
C3	0.0306 (15)	0.0362 (16)	0.0339 (14)	−0.0062 (13)	0.0182 (13)	−0.0017 (12)
C4	0.050 (3)	0.086 (4)	0.073 (3)	−0.026 (3)	0.049 (3)	−0.037 (3)
C5	0.0329 (17)	0.051 (2)	0.0396 (17)	−0.0085 (15)	0.0229 (15)	−0.0123 (15)
C6	0.0372 (18)	0.0422 (19)	0.0367 (15)	0.0006 (15)	0.0196 (14)	0.0103 (14)
C7	0.0289 (15)	0.0321 (15)	0.0351 (14)	−0.0015 (12)	0.0175 (12)	0.0000 (12)
C8	0.0299 (15)	0.0305 (14)	0.0344 (13)	0.0002 (12)	0.0178 (12)	−0.0008 (11)
C9	0.044 (2)	0.0376 (17)	0.0414 (17)	−0.0063 (15)	0.0254 (16)	−0.0054 (14)

### Geometric parameters (Å, °)

**Table:**

Re1—C3	1.905 (4)	O3—C3	1.155 (4)
Re1—C2	1.914 (4)	O4—C8	1.257 (4)
Re1—C1	1.924 (3)	O5—C8	1.227 (4)
Re1—N2	2.135 (3)	N1—C7	1.147 (4)
Re1—N1	2.138 (3)	N2—C5	1.136 (5)
Re1—O4	2.153 (2)	C4—C5	1.454 (6)
F1A—C9	1.354 (8)	C4—H4A	0.9800
F2A—C9	1.253 (7)	C4—H4B	0.9800
F3A—C9	1.285 (9)	C4—H4C	0.9800
F1B—C9	1.296 (8)	C6—C7	1.444 (5)
F2B—C9	1.356 (9)	C6—H6A	0.9800
F3B—C9	1.241 (10)	C6—H6B	0.9800
O1—C1	1.143 (4)	C6—H6C	0.9800
O2—C2	1.149 (4)	C8—C9	1.541 (5)
			
C3—Re1—C2	89.26 (15)	H6A—C6—H6B	109.5
C3—Re1—C1	89.64 (14)	C7—C6—H6C	109.5
C2—Re1—C1	88.16 (15)	H6A—C6—H6C	109.5
C3—Re1—N2	94.75 (13)	H6B—C6—H6C	109.5
C2—Re1—N2	93.44 (14)	N1—C7—C6	178.2 (4)
C1—Re1—N2	175.34 (12)	O5—C8—O4	129.8 (3)
C3—Re1—N1	92.03 (13)	O5—C8—C9	116.0 (3)
C2—Re1—N1	175.23 (12)	O4—C8—C9	114.2 (3)
C1—Re1—N1	96.43 (13)	F3B—C9—F2A	128.8 (8)
N2—Re1—N1	81.88 (12)	F3B—C9—F3A	36.0 (9)
C3—Re1—O4	173.21 (12)	F2A—C9—F3A	111.5 (9)
C2—Re1—O4	96.39 (13)	F3B—C9—F1B	108.5 (9)
C1—Re1—O4	94.28 (11)	F2A—C9—F1B	57.9 (7)
N2—Re1—O4	81.20 (10)	F3A—C9—F1B	130.9 (7)
N1—Re1—O4	82.04 (10)	F3B—C9—F1A	69.5 (10)
C8—O4—Re1	122.0 (2)	F2A—C9—F1A	106.7 (7)
C7—N1—Re1	170.6 (3)	F3A—C9—F1A	104.8 (8)
C5—N2—Re1	176.2 (3)	F1B—C9—F1A	50.1 (7)
O1—C1—Re1	178.5 (3)	F3B—C9—F2B	103.4 (10)
O2—C2—Re1	178.7 (4)	F2A—C9—F2B	46.1 (7)
O3—C3—Re1	178.3 (3)	F3A—C9—F2B	71.6 (10)
C5—C4—H4A	109.5	F1B—C9—F2B	101.3 (9)
C5—C4—H4B	109.5	F1A—C9—F2B	140.3 (6)
H4A—C4—H4B	109.5	F3B—C9—C8	116.3 (6)
C5—C4—H4C	109.5	F2A—C9—C8	113.1 (5)
H4A—C4—H4C	109.5	F3A—C9—C8	111.8 (6)
H4B—C4—H4C	109.5	F1B—C9—C8	116.1 (4)
N2—C5—C4	178.6 (5)	F1A—C9—C8	108.4 (4)
C7—C6—H6A	109.5	F2B—C9—C8	109.4 (5)
C7—C6—H6B	109.5		
			
C2—Re1—O4—C8	−43.2 (3)	O4—C8—C9—F2A	−72.6 (9)
C1—Re1—O4—C8	45.4 (3)	O5—C8—C9—F3A	−19.7 (11)
N2—Re1—O4—C8	−135.7 (3)	O4—C8—C9—F3A	160.5 (10)
N1—Re1—O4—C8	141.3 (3)	O5—C8—C9—F1B	171.4 (10)
Re1—O4—C8—O5	1.2 (5)	O4—C8—C9—F1B	−8.3 (11)
Re1—O4—C8—C9	−179.1 (2)	O5—C8—C9—F1A	−134.7 (7)
O5—C8—C9—F3B	−59.0 (13)	O4—C8—C9—F1A	45.5 (8)
O4—C8—C9—F3B	121.3 (13)	O5—C8—C9—F2B	57.6 (11)
O5—C8—C9—F2A	107.1 (9)	O4—C8—C9—F2B	−122.1 (10)

### Hydrogen-bond geometry (Å, °)

**Table:**

*D*—H···*A*	*D*—H	H···*A*	*D*···*A*	*D*—H···*A*
C4—H4B···O5^i^	0.98	2.42	3.113 (5)	127.
C6—H6A···F1A^ii^	0.98	2.35	3.237 (8)	150.
C6—H6B···O5^iii^	0.98	2.46	3.075 (5)	121.

Symmetry codes: (i) −*x*+1, *y*−1/2, −*z*+3/2; (ii) −*x*+2, *y*−1/2, −*z*+5/2; (iii) −*x*+2, −*y*, −*z*+2.
